# De Novo Status Epilepticus in patients with COVID‐19

**DOI:** 10.1002/acn3.51071

**Published:** 2020-06-10

**Authors:** Sana Somani, Sandipan Pati, Tyler Gaston, Alissa Chitlangia, Shruti Agnihotri

**Affiliations:** ^1^ Department of Neurology University of Alabama at Birmingham Birmingham AL USA

## Abstract

Neurological complications are increasingly recognized with SARS‐CoV‐2, the causative pathogen for COVID‐19. We present a single‐center retrospective case series reporting the EEG and outcome of de novo status epilepticus (SE) in two African‐American women with laboratory‐confirmed SARS‐CoV‐2 virus. SE was the initial presentation in one asymptomatic individual. Patient 2 had COVID‐19 pneumonia, and fluctuating mental status that raised the suspicion of subclinical SE. The patient with older age and higher comorbidities failed to recover from the viral illness that has no definitive treatment.

## Introduction

With the emergence of the COVID‐19 pandemic, there has been a surge in reporting clinical phenotype, outcome, and treatment options against the pathogen – a novel coronavirus named SARS‐CoV‐2.^1^, ^2^ While fever, respiratory distress, and gastrointestinal disturbances have been some of the common presenting symptoms, reports of neurological manifestations are limited.^3^ The goal of this case series is to report the EEG, clinical findings, and outcome of de novo status epilepticus (i.e., new‐onset SE without a prior history of seizure or epilepsy) in two patients with COVID‐19. Incidentally, both patients are African Americans (AA) – a cohort that has higher mortality from COVID‐19.^4^

## Patients and Methods

This is a single‐center retrospective case series of de novo SE in AA women with laboratory‐confirmed SARS‐CoV‐2.

### Case 1

**Figure 1:**
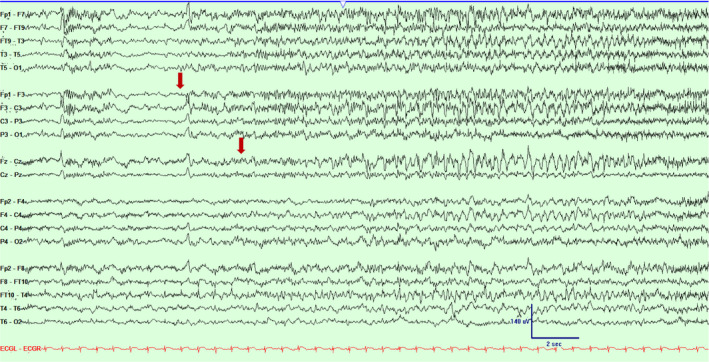
Scalp EEG in a bipolar montage from subject #1 demonstrating emergence of low amplitude ictal fast rhythmic activity over left fronto‐central and midline regions (marked with an arrow). Timebase 15 mm/sec, sensitivity 5 uV/mm, LFF 1 Hz, HFF 70 Hz.

**Figure 2:**
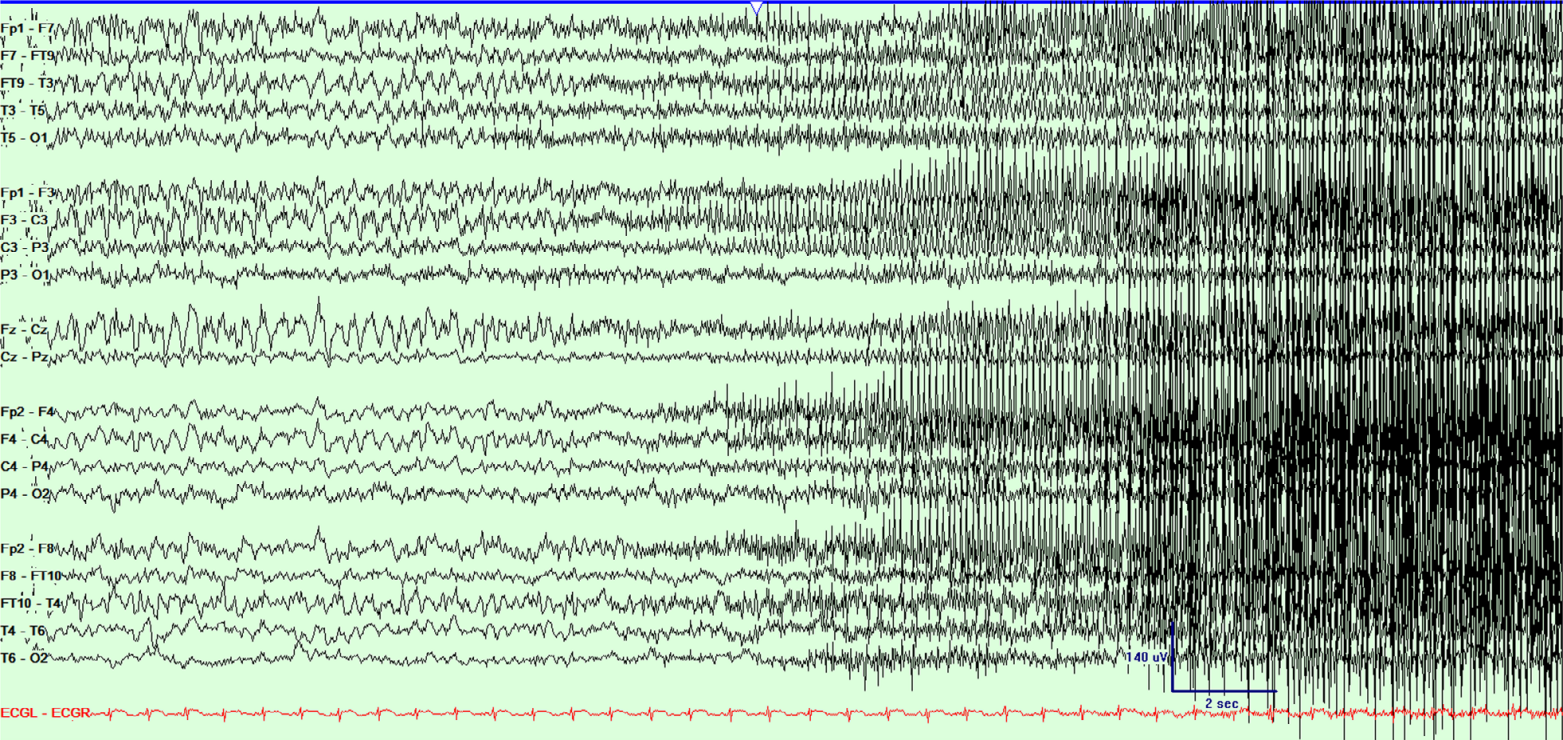
Scalp EEG in a bipolar montage from subject #1 demonstrating the progression of the seizure (Fig. 1) and clinical accompaniment with bilateral tonic–clonic activity. Timebase 15 mm/sec, sensitivity 5 uV/mm, LFF 1 Hz, HFF 70 Hz.

A 49‐year‐old right‐handed AA woman with a past medical history of rheumatoid arthritis, schizoaffective, and conversion disorder presented to the UAB emergency room for evaluation of altered mental status after a witnessed seizure. There was no travel or contact exposure to COVID‐19, and she had no symptoms of COVID‐19. At presentation, she had an axillary temperature of 99.5F, respiratory rate of 16 breaths per minute, heart rate of 120 beats per minute, blood pressure of 180/95 mm Hg, and oxygen saturation of 94%. The neurologic exam revealed lethargy and disorientation to time and place. There was no meningismus. CT head was normal. Continuous video EEG monitoring was obtained to rule out subclinical seizures and status epilepticus. Scalp EEG showed severe background slowing in the delta range and the presence of multiple seizures (approx. 4‐6 every hour over the next 8 hours) emanating from the midline and left fronto‐central regions (Figs. 1, 2). Initially, there was a clinical correlate of facial twitching, head version to the right followed by a bilateral tonic–clonic seizure. In the latter half, there was electroclinical dissociation with the presence of only electrographic seizures. The patient was intubated and treated with multiple doses of lorazepam, levetiracetam, and propofol for sedation. Soon after arrival, the patient was tested for SARS‐CoV‐2 by reverse‐transcriptase polymerase chain reaction (RT‐PCR) through a nasal swab specimen, which returned negative. MRI brain without contrast was unrevealing for any definitive pathology. Her clinical course over the next 36 hours improved significantly with resolution in status epilepticus, and she was extubated within 48 hours. A lumbar puncture was not performed due to rapid improvement, and excellent response to levetiracetam. However, within 72 hours of the initial presentation, the patient developed fever, shortness of breath, myalgias, and nonproductive cough. A repeat nasal swab RT‐PCR for SARS‐CoV‐2 returned positive. She remained stable with a mild cough and was eventually discharged home on levetiracetam and recommendation to self‐quarantine.

### Case 2

**Figure 3:**
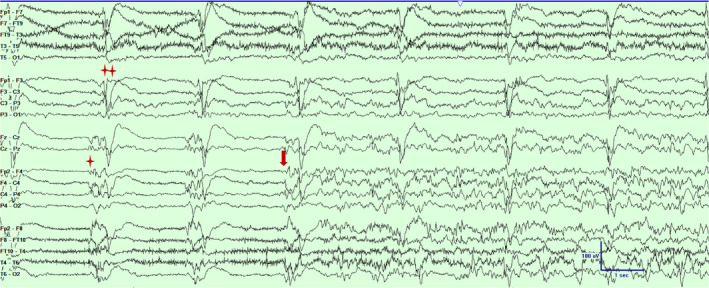
Scalp EEG in a bipolar montage from subject #2 demonstrating independent periodic discharges over left (marked with double asterisks) and the right hemisphere (marked with an asterisk) and a focal seizure emanating from the right fronto‐central‐parietal region (marked with an arrow).Timebase 30mm/sec, sensitivity 5 uV/mm, LFF 1 Hz, HFF 70 Hz.

**Figure 4:**
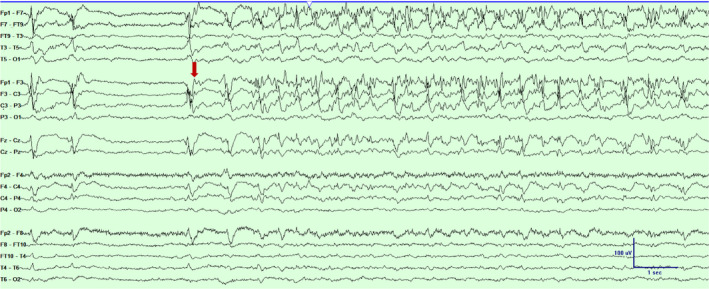
Scalp EEG in a bipolar montage from subject #2 demonstrating a focal seizure emanating from the left fronto‐central‐parietal region (marked with an arrow). Timebase 30mm/sec, sensitivity 5 uV/mm, LFF 1 Hz, HFF 70 Hz.

A 73‐year‐old right‐handed AA woman with a known history of hypertension, diabetes mellitus, and chronic kidney disease presented to the UAB emergency room with shortness of breath, lower extremity edema, and confusion. There was no travel history, and contact exposure to COVID‐19 was unknown. A significant past neurological history included surgical repair of left frontal skull base ethmoidal encephalocele and ventriculoperitoneal shunt for hydrocephalus. At presentation, she had an axillary temperature of 90.6 F, heart rate of 44 beats per minute, blood pressure of 86/71 mm Hg, and oxygen saturation of 100%. There was no focal neurological weakness or neck stiffness, but she had diminished level of consciousness. Chest X‐ray demonstrated patchy bilateral opacities and serum procalcitonin (0.53 ng/mL) was elevated. She was admitted to the intensive care unit with the diagnosis of sepsis. The next morning, bedside nurses noticed persistent face and arm myoclonus with worsening altered mental status that was concerning for status epilepticus. Scalp EEG showed marked background voltage attenuation and slowing, continuous 0.5‐0.75 Hz bilateral independent periodic discharges (BIPDs) over the left and right hemisphere that evolved to form recurrent (approx. 5/hour) discrete seizures emanating from either right or left fronto‐central‐parietal regions (Figs. 3, 4). Myoclonic status epilepticus with coma (MSE) was diagnosed and treated with intravenous levetiracetam, lacosamide, phenytoin, and midazolam to achieve burst suppression for 48 hours. Four days later, the EEG improved significantly with the cessation of epileptiform activity. However, clinically she worsened with multi‐organ failure requiring blood transfusions and hemodialysis. Tracheal aspirate tested positive for SARS‐CoV‐2 by RT‐PCR. Due to a strict isolation policy to minimize the spread of COVID‐19, the MRI brain was not obtained. Instead, a CT brain and perfusion study were performed a day after the MSE ceased, and it did not reveal any abnormality that could explain the etiology of MSE. For the next 3 weeks, multiple attempts to arouse or extubate failed after sedatives were held. Care was withdrawn, and the patient expired.

## Discussion

SE without a prior history of seizure or epilepsy is rare, although reported in the presence of autoimmune, inflammatory, and infective encephalitis.^5^ Both patients had confirmed SARS‐CoV‐2 pneumonia (Table 1), but we were unable to confirm encephalitis as CSF analysis was not performed. Patient #1 had no febrile illness or myalgias at presentation, but the subclinical nature of COVID‐19 is increasingly recognized,^6^ and it underscores the importance of universal testing in all patients admitted to the hospital. The sensitivity of rRT‐PCR has also been reported to be variable, ranging from 59% in throat swab samples^7^ to 83.3% in other studies.^8^ The initial false‐negative rRT‐PCR from the nasopharyngeal swab of Patient #1 can be due to variable viral shedding; lower test sensitivity observed early in the disease,^9^ immature development of nucleic acid detection technology or improper clinical sampling.^7^ It has been reported that for all stages of the disease, the highest positive detection rate has been with sputum swabs followed by nasal swabs.^10^ The possibility of being infected in the hospital is less likely, considering the febrile illness started within 72 hours of admission (incubation period is typically 5 days).

**Table 1:** Clinical summary of the two patients with laboratory‐confirmed SARS‐CoV‐2.

	Patient #1	Patient #2
Age, Sex	49, F	73, F
Significant comorbidities	BMI 33.6 kg/m^2^ Rheumatoid arthritis Schizoaffective disorder	BMI 38.2 kg/m^2^ End‐stage kidney disease (on hemodialysis) Uncontrolled DM Skull base encephalocele, repaired with VP shunt
Preadmission medications	Celecoxib paliperidone	aspirin, amlodipine, insulin, lovastatin, carvedilol, sevelamer, furosemide
COVID‐19 symptoms at presentation	Asymptomatic Chest X ray – normal	Respiratory distress Chest X‐ray patchy bilateral opacities
Significant neuroimaging findings	MRI brain – NA	CT brain and perfusion study – NA
EEG findings	Background – delta slowing Interictal – none Ictal – frequent (4‐6/hour) cyclical seizures emanating from left fronto‐central regions	Background – very low voltage, 1‐2 Hz activity Interictal – 0.5‐0.75 Hz bilateral independent periodic discharges Ictal – frequent (5/hour) cyclical seizures emanating from left and right fronto‐central regions
Treatment of Status epilepticus	lorazepam levetiracetam	lorazepam, levetiracetam, lacosamide, phenytoin, midazolam for BS
Outcome	SE stopped, extubated, survived	SE stopped, failed extubation, died

Abbreviations: BMI, body mass index; BS, burst suppression; NA, no abnormality; SE, status epilepticus.

Patient #2 had new‐onset refractory status epilepticus (NORSE). NORSE is a clinical presentation and not a diagnosis that is reserved for patients without any – (a) history of epilepsy; (b) absence of any definite structural or metabolic cause of SE; and (c) failed one first line and one second‐line medication.^11^ Viral genomic analysis of SARS‐CoV‐2 confirmed homologous sequences shared with SARS‐CoV‐1.^12^ The neurovirulence of SARS‐CoV‐1 is established from the human autopsy series that confirmed the presence of viral antigen in the hippocampus, thalamus, medulla, and other brainstem regions that regulate cardiorespiratory functions.^13^ The mode of transmission to the brain could be either through systemic circulation during viremia or a transneuronal propagation through the upper nasal transcribrial route.

## Conclusion

De novo SE can be the initial presentation in asymptomatic COVID‐19. Altered mental status in patients with COVID‐19 pneumonia can be due to SE. The presence of significant comorbidities can determine survival from the viral illness that has no definitive treatment yet.

## Conflict of Interest

None.
